# Generation of intense quasi-electrostatic fields due to deposition of particles accelerated by petawatt-range laser-matter interactions

**DOI:** 10.1038/s41598-019-44937-2

**Published:** 2019-06-12

**Authors:** F. Consoli, R. De Angelis, T. S. Robinson, S. Giltrap, G. S. Hicks, E. J. Ditter, O. C. Ettlinger, Z. Najmudin, M. Notley, R. A. Smith

**Affiliations:** 1 ENEA – C.R. Frascati, Fusion and Nuclear Safety Department, Via E. Fermi 45, 00044 Frascati, Italy; 2 The Blackett Laboratory, Imperial College London, Prince Consort Road, London, SW7 2AZ United Kingdom; 3 Central Laser Facility, STFC Rutherford Appleton Laboratory, Chilton, Didcot, Oxon OX11 0QX United Kingdom

**Keywords:** Applied optics, Applied physics, Laser-produced plasmas, Nuclear fusion and fission, Electrical and electronic engineering

## Abstract

We demonstrate here for the first time that charge emitted by laser-target interactions at petawatt peak-powers can be efficiently deposited on a capacitor-collector structure far away from the target and lead to the rapid (tens of nanoseconds) generation of large quasi-static electric fields over wide (tens-of-centimeters scale-length) regions, with intensities much higher than common ElectroMagnetic Pulses (EMPs) generated by the same experiment in the same position. A good agreement was obtained between measurements from a classical field-probe and calculations based on particle-flux measurements from a Thomson spectrometer. Proof-of-principle particle-in-cell simulations reproduced the measurements of field evolution in time, giving a useful insight into the charging process, generation and distribution of fields. The understanding of this charging phenomenon and of the related intense fields, which can reach the MV/m order and in specific configurations might also exceed it, is very important for present and future facilities studying laser-plasma-acceleration and inertial-confinement-fusion, but also for application to the conditioning of accelerated charged-particles, the generation of intense electric and magnetic fields and many other multidisciplinary high-power laser-driven processes.

## Introduction

The generation of particle beams due to the interaction of energetic, high-intensity, short-pulse lasers with a target is currently an important topic of research^1^ with the potential to underpin future table-top and high-peak current particle accelerators. There are however currently limitations to the use of these compact high-flux sources for potential applications, particularly due to their intrinsic broad-energy spectrum and energy-dependent spatial divergence. Thus, many investigations have been focused on overcoming these constraints^1–9^ with the aim of extending the range of possible applications of laser-driven accelerators to radiography^10,11^, oncology^12,13^, medical imaging^14^, astrophysics^15,16^, high-energy-density-physics^17,18^ and ion-beam fast-ignition in the frame of inertial confinement fusion^19^. In addition, the large charge-separation induced over a few picosecond timescale in these experiments can be very interesting for uses quite different and distinct from classical particle beam - target schemes. Tailored large-amplitude electric and magnetic fields can also be efficiently created in this way^2,6,9,20–24^. When high intensity lasers are applied to a target, electrons are accelerated first, typically to relativistic energies^1,25,26^. If the interaction is on the surface of a suitable hollow-cylinder target, an ultrafast laser-driven electrostatic micro-lens can be created^2,6^, with very high transient radial *electric fields*, capable of focusing and energy-selecting MeV-range proton beams produced by an accelerator placed nearby, either one of a classical type or one based on laser-matter interactions. Alternatively, very strong quasi-static *magnetic fields* can be generated from the interaction of lasers with spiral targets^24^ or target-coil geometries^20–23,27^, and used for similar beam conditioning purposes. Once fast electrons leave the target, they are followed later in time by the comparatively much slower laser-accelerated ions. An overall positive charge is thus present on the target for relatively large times^25,26,28,29^. In a conductive target setup, intense transient currents are thus spontaneously created as a result; they act to neutralize this charge, and are capable of driving powerful electromagnetic waves^9,28–32^. The transient fields generated by such charge-waves can be very effective for collimation and post-acceleration of laser-driven ion beams, for example in coil-shaped targets^9^. On the other hand, these transient currents are also known to be one of the main sources of intense *ElectroMagnetic Pulse* (*EMP*) generation in the radiofrequency-microwave range^28,29,32^, whose field is capable of degrading data capture or damaging electronic equipment close to the experimental chamber. In all these cases, the region where the generated fields have maximum intensity is mainly localized to a rather small volume around the laser interaction-point on the target, which is thus also heavily affected by ionizing electromagnetic (UV, X and γ rays) and particle radiation coming from the laser-plasma. Although there is now a strong interest in studying and employing these schemes of electric and magnetic field generation, one of the associated well-known problems is the difficulty of performing reliable and accurate field measurements^2,20–22^, since the local environment of a laser-matter interaction (with high particle and radiation fluxes, strong shocks and plasma flow) places severe limitations. These extreme conditions are also a considerable challenge for many proposed applications of these electromagnetic fields^2,6,9,20–24,33^ and this will be an increasing problem in the future with the advent of higher repetition-rate laser drivers and the shift in focus from proof of principle applications to routine operation.

**Figure 1:**
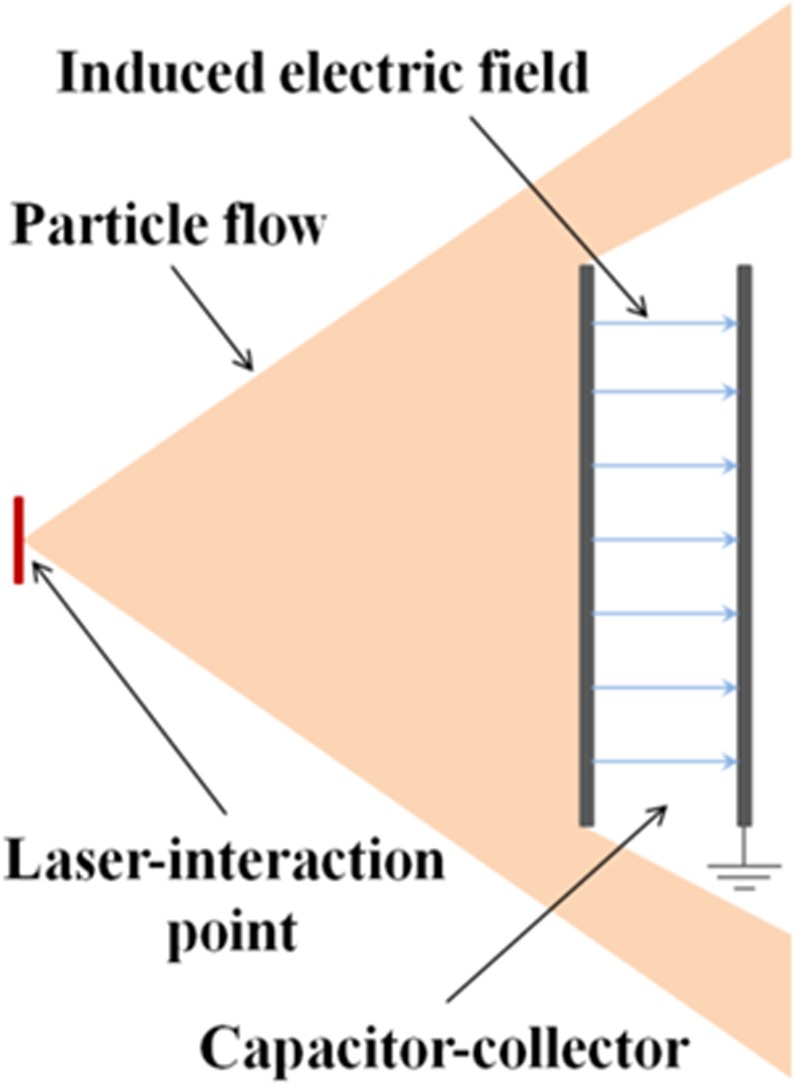
Principle scheme of the field induced due to charge deposition on one plate of a capacitor-collector setup. The system is initiated by an energetic particle flow from a pulsed laser driven source, impulsively creating a large electric field, while also providing shielding from direct ionizing radiation.

In this work we show for the first time that the charge emitted by an intense laser-target interaction can be efficiently deposited onto one plate of an open capacitor-collector structure (see the basic scheme of Fig. 1) and can thus be used for the fast generation of very large quasi-static electric fields. These fields can be easily obtained in regions having large spatial extent and which can be well shielded against direct ionizing-radiation produced by the powerful laser-matter interaction. Simple evolutions from the basic scheme shown in Fig. 1, can also readily lead to more complex structures where specific electric field distributions, not necessarily uniform, can be achieved. Due to the extreme laminarity of laser-driven particle flows^1^, it is possible to place the charge-collector plate at some distance from the laser interaction-point, while enlarging its transverse dimension to ensure capture of the majority of the particle stream. Increasing the distance in this way has the advantage of reducing the intensity of unwanted ionizing electromagnetic radiation from the plasma reaching the collector. Moreover, the collection plate can be made of high-atomic-number materials thick enough to provide suitable shielding of the electric-field-area behind it, where field measurements or applications can be conducted. This is one of the main advantages of this simple setup.

Depending on the position and geometry of the collector, these powerful electric fields can have intensities much higher than the EMPs generated in the same experiment. In particular, this is possible at locations far away from the target, where EMP intensity decreases^28,29,32^. As shown in this paper, their accurate measurement can be performed by classical field-probes, and the schemes for their production can be efficiently run with repetitive high-power lasers. One application of primary importance for them is the efficient focusing, deflection and energy-selection of the charged particle beams emitted by a separate apparatus^34^, such as a conventional accelerator or one based on a laser-plasma interaction. The large deposited charge leads to remarkable electric fields and related applied voltages, with the important characteristic that they can be created with short rise-times and at a desired time instants absolutely synchronized with the main laser beam, something very difficult to obtain with a conventional power supply. This is of great interest for the application of these fields to charged particles accelerated by laser-matter interaction, with laser pulse generated by the same main oscillator. Moreover, very high currents can be generated if the large accumulated charge is then short-circuited to ground by a fast switch. This can be used to drive ultrashort travelling electromagnetic waves for controlling and optimizing laser-accelerated ions^9^, or to generate very high magnetic fields that can be suitable for many different purposes^4,5,8,16,20–24,27,33^. Improvements to particle-acceleration schemes are of immediate interest for the phenomenon described, but the remarkable features of the technique also lends itself to a range of further applications that is very wide and multidisciplinary. In particular, these electric fields of high intensity and wide spatial extent can be directly applicable for biological and medical studies^35^, material and device characterization^36–38^, electromagnetic-compatibility investigations^38^ and terahertz generation^39,40^.

## Results

### Experimental measurements

**Figure 2:**
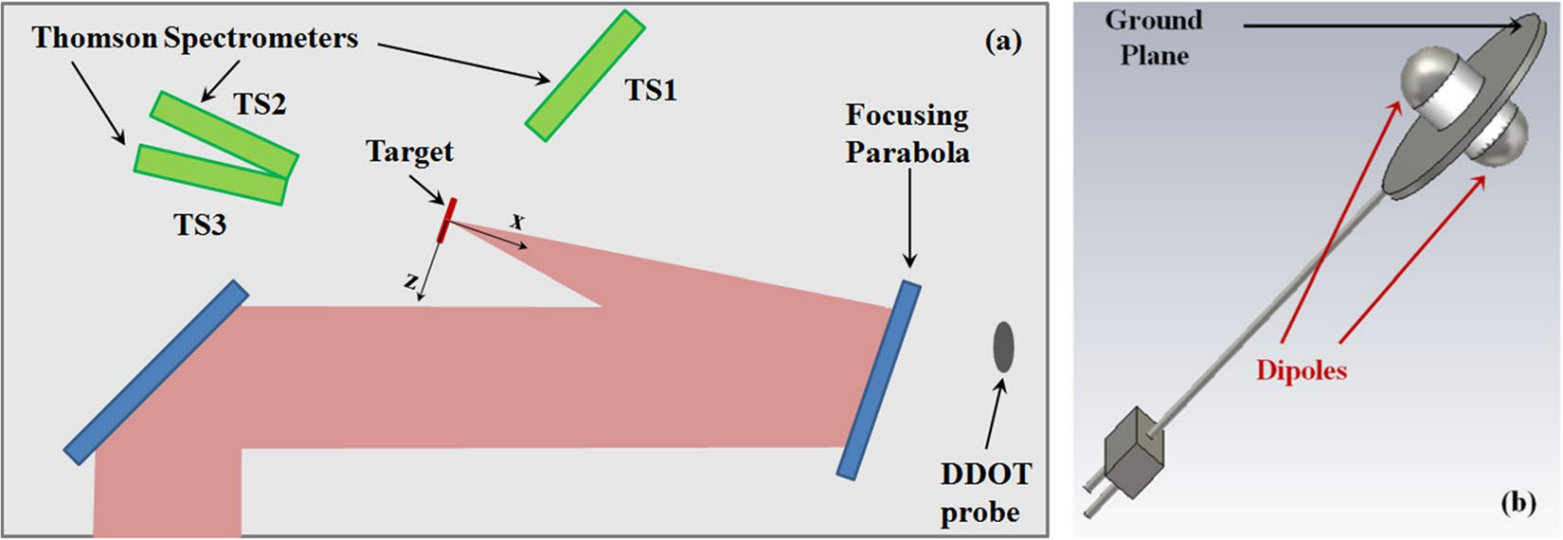
(**a**) Top-view scheme of the vacuum chamber; ~400 J of laser energy (red beam) is brought to a ∼6 μm FWHM focal-spot diameter on a thin-foil target using an F/3 off-axis parabola, with the resulting particle flux diagnosed by multiple Thomson ion spectrometers. (**b**) Scheme of the D-Dot probe used, situated behind the parabola.

The geometry of the setup used in the experiment is shown in Fig. 2a. A laser pulse is focused by an off-axis parabolic mirror onto a thin plastic target at normal incidence. The coordinate system we used to describe the geometry is centered on the laser-target interaction point, with the *x-*coordinate normal to target surface. Three Thomson Spectrometers were used to detect particles emitted by means of TNSA process^1^ along forward (TS2 and TS3) and backward (TS1) directions with respect to the incoming laser. The focusing parabola consists of a 110 mm thick, 650 mm diameter glass substrate with a 620 mm diameter silver front-surface, placed at ∼1.8 m distance from target. A D-Dot differential electric-field sensor^41^ (see Fig. 2b) was placed behind the parabola (as shown in Fig. 2a) providing good isolation from direct particle and ionizing electromagnetic radiation fluxes from the target, as discussed in the Methods section.

**Figure 3:**
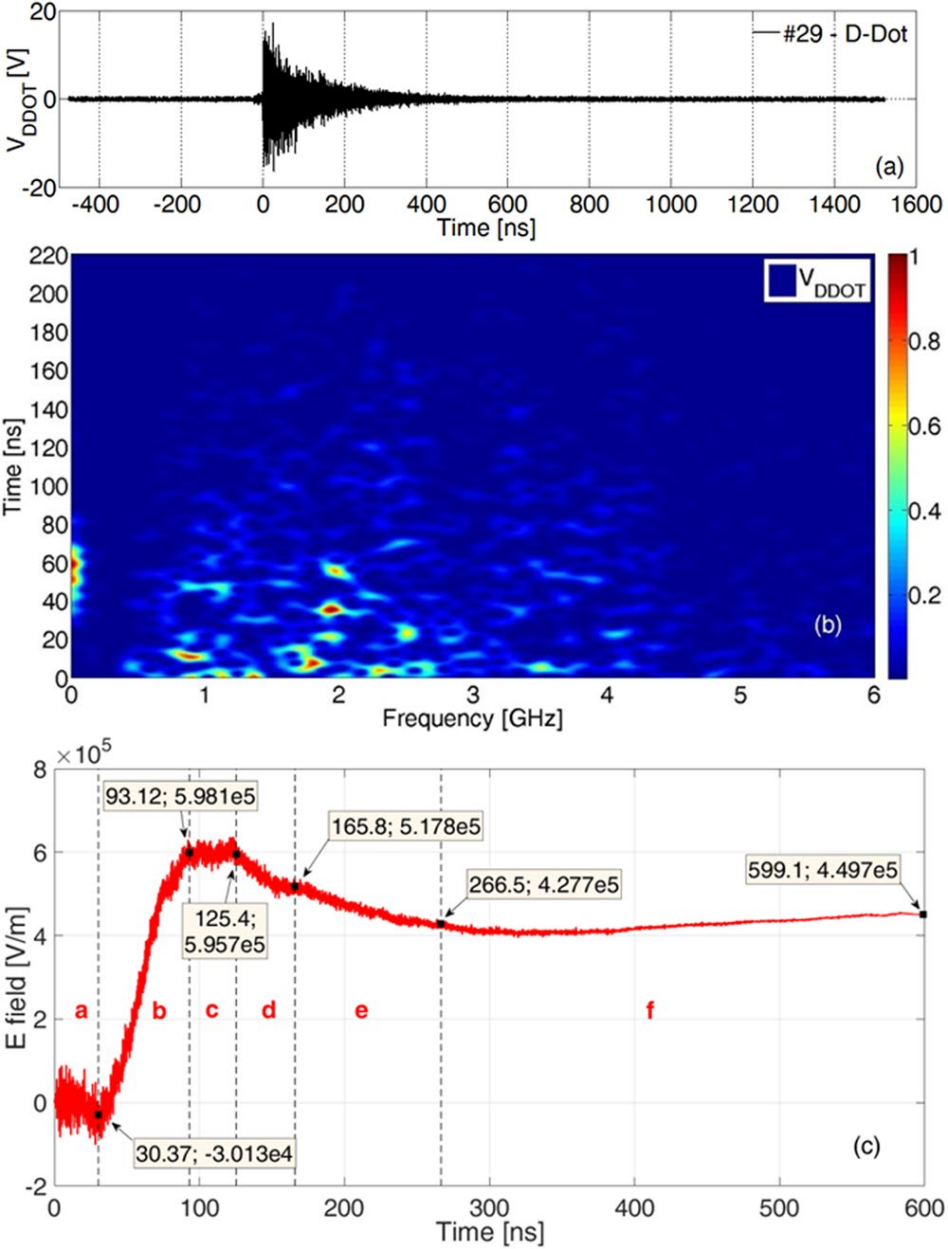
(**a**) *V*_*DDOT*_ signal detected by the D-Dot probe in shot #29; (**b**) time-gated normalized spectrogram of the signal; (**c**) recovered component of the electric field normal to the D-Dot ground plane. Time intervals *a-f* identified in (**c**) are discussed in more detail in the main text. In this Figure and in the next Fig. 4 the origin of the time scale was set at the moment when the EMP signal reaches the D-Dot probe.

**Figure 4:**
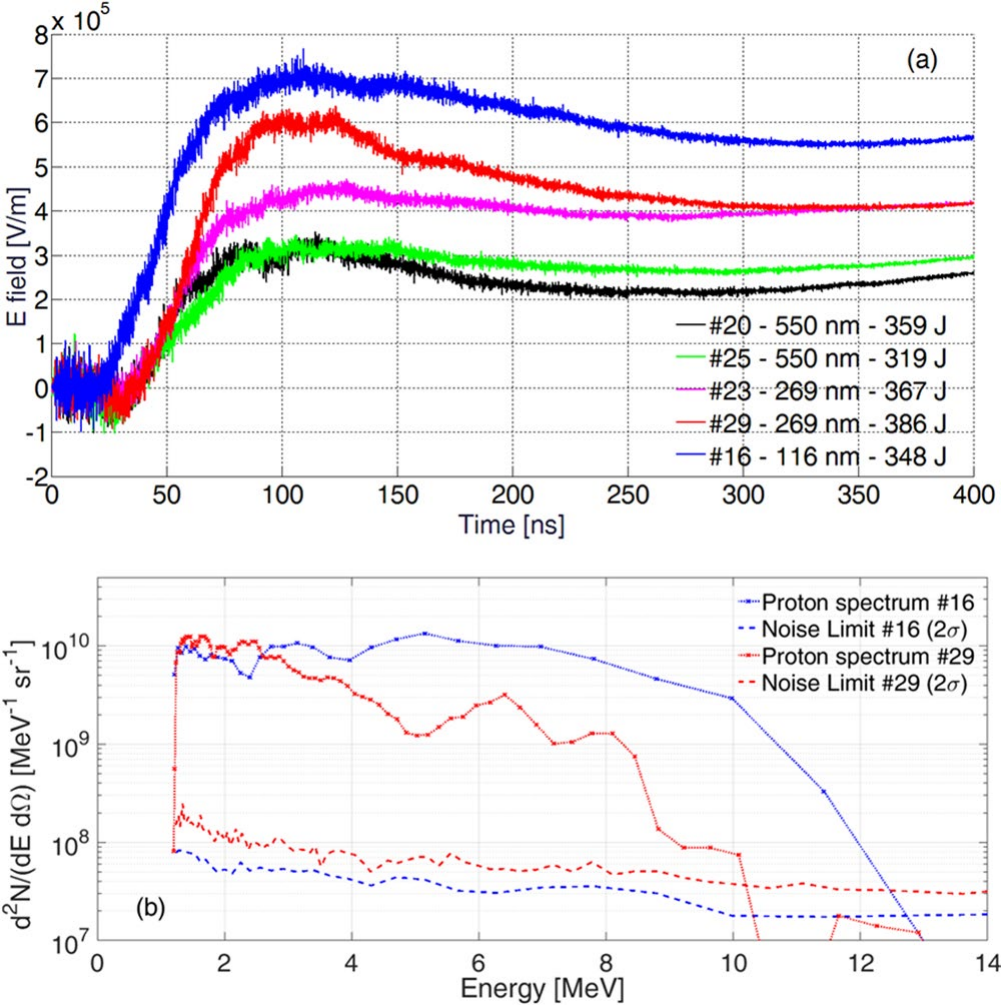
(**a**) Comparison of several single-shot measurements of the electrical field component normal to the D-Dot ground plane; (**b**) spectra of the detected proton flux measured by Thomson Spectrometer TS1 for different shots and comparison of data and detection Noise Limit for #16 with 348 J laser energy, 1.22 ps pulse duration, 1.08 × 10^21^ W/cm^2^ intensity on a Parylene-N target of 116 nm thickness (see Table 1) and for #29 with 386 J laser energy, 1.70 ps pulse duration, 4.8 × 10^20^ W/cm^2^ intensity on a Parylene-N target of 269 nm thickness.

A differential signal is generated across the D-Dot probe, proportional to the time-derivative of the incident electric-field component normal to the probe ground-plane. A *balun*^41^ was connected to the two probe outputs to convey this signal to a long coaxial cable coupled to a fast oscilloscope. The whole configuration provided more than 40 dB of common-mode-signal rejection for frequencies up to 200 MHz and 28 dB up to 6 GHz. For a 386 J, 1.7 ps laser pulse on target (shot #29, see Table 1 for details) the resulting *V*_*DDOT*_ signal is shown in Fig. 3a. At first sight, the trace looks like a classical *ElectroMagnetic Pulse* generated by laser-plasma interaction^42–48^, with a fast rise followed by an envelope with an exponential decay. It is well known from the literature^28,29,32,48,49^ that a strong EMP signal is generated at the moment of laser-target interaction. As described in the Methods section, we verified with nanosecond accuracy that this occurred also in this experiment and that the EMP thus reached the D-Dot probe at light velocity, after a propagation time *t*_*s*_ = *d*_*DDOT*_/*c ∼* 7.4 ns, being *d*_*DDOT*_ the D-Dot distance from target and *c* the speed of light in vacuum. We found it convenient for Figs 3 and 4 to use a time-scale where the origin was set at the moment when the EMP signal generated at the target first reached the D-Dot probe. This means that in this shifted time-scale the laser pulse arrived to the target at the instant *t* = *−t*_*s*_.

**Table 1:** Shots parameters.

#	Target Thickness [nm]	Energy [J]	Duration [fs]	Intensity [10^20^ W/cm^2^]
20	550	359	980	14.3
25	550	319	1000	10.9
23	269	367	—	—
29	269	386	1700	4.8
16	116	348	1220	10.8

No measurement of pulse duration were obtained for #23, where the focal spot area was of the order of 4 × 10^−7^ cm^2^. For a reasonable pulse width in the (980,1700) fs interval, we can estimate a peak intensity in the (5.4,9.4) × 10^20^ W/cm^2^ range.

In Fig. 3a it is shown that the time duration of the signal, with respect to the background electrical noise of the oscilloscope, is ∼400 ns. The time-resolved spectrogram^46^ of this signal is shown in Fig. 3b. It is apparent that a low-frequency component (LFC) is present approximately over the (40,80) ns interval, while the remaining high-frequency component (HFC) has a broad spectrum up to at least 6 GHz.

Through a process of accurate cable frequency-domain deembedding, described in the next Methods section, from the *V*_*DDOT*_ signal it was possible to recover the actual *V*_*DDOT-B*_ produced at the balun output. The component of the applied electric field normal to the D-Dot ground plane can be thus found by time integration of *V*_*DDOT-B*_^41^, and is shown for #29 in Fig. 3c. The two main components, already identified with the aid of the spectrogram, are here rather clear. In particular, the broadband HFC appears as a modulation with respect to the low-frequency component; it has a maximum peak-to-peak amplitude Δ*E*_*HFC-pp*_ ∼ 172 kV/m, visible in the (0,30) ns time interval (named *a* in the picture) and gradually decreases with time. This is the classical form of an *EMP* due to a laser-plasma interaction^32,42–48^. Very interesting results are visible for the LFC. A high field increase on the (30,93) ns time interval (named *b* in Fig. 3c) is visible: Δ*E*_*LFC*_ ∼ 600 kV/m, that we were able to detect with good accuracy, as described in detail in the Methods section. In particular, it results ${\rm{\Delta }}{E}_{LFC}/({\rm{\Delta }}{E}_{HFC-pp}/2)\approx 7$. There is a noticeable slow decrease of the field intensity over the *d* and *e* time-intervals. Signal variations are rather low over intervals *a*, *c* and *f*, and comparable with the related measurement accuracy.

As discussed, the origin of the time-scale in Fig. 3c is delayed by the *t*_*s*_ propagation time with respect to the moment when the laser-pulse hits the target. It is thus evident that the rise of the huge field observed in the *b* interval (Fig. 3c), clearly delayed with respect to the moment when the laser hits the target, has to be related to charged particles accelerated by the laser-matter interaction as distinct from prompt electromagnetic radiation. In particular, it can be readily associated with charges reaching the silver-coated glass mirror of the focusing parabola and depositing on its surface. The small slopes of the *c*, *d*, *e*, *f* time intervals in Fig. 3c suggest that some neutralization of the deposited charge on the parabola then occurs, which might come from either other particles arriving at later times, or charge relaxation-processes with a time-constant governed by the parabola structure, its supporting frame, and the equivalent complex-impedance modeling the whole chamber and all the objects within it. This would finally lead to the slow decay to zero of the field.

The laser acceleration mechanism in our experiments lies in the target-normal sheath acceleration (TNSA) regime^1^, and under these conditions energetic multi-MeV ions are expected in the forward direction (and observed by the Thompson spectrometers TS2 and TS3 shown in Fig. 2; a paper is in preparation regarding these results). It is well known that these forward ions are accompanied by particles accelerated in the backward direction (towards the laser) and with a broad angular distribution^1,50–54^. In particular, in some conditions of high focal intensities on sub-micrometer targets it has been shown that forward and backward accelerated ions can have roughly comparable energies^50,52–54^. If we consider the simple scheme of a classical time-of-flight detector, from geometrical considerations we can estimate the energy range of possible particles reaching the parabola surface from the extremes of the *b* time-interval in Fig. 3: electrons up to keV energy and protons up to MeV energy. In Fig. 4a we show the comparison of multiple electric field profiles obtained by D-Dot measurements when shooting with similar laser energy on targets made of the same plastic but of different thicknesses, as detailed in Table 1. Higher fields are generated for targets with smaller thickness. Indeed, this is also the condition to achieve more accelerated particles and at higher energy^1,50,53,54^. The timing of the rise of the electric field relative to the time of the laser pulse on target also changes depending on the shot; in particular, for the thinnest target (shot #16) this occurs earlier with respect to the others, as expected for more energetic protons. These considerations are well confirmed by the spectrum of protons detected by the TS1 spectrometer along the backward direction (see Fig. 2a), given for #16 and #29 in Fig. 4b together with their associated detection noise-limits. Protons with energies exceeding 10 MeV were detected. From these spectra we estimated the overall proton flux per solid angle dN_p_/dΩ and the associated particle energy along the direction of the TS1 spectrometer: 7.5 × 10^10^ ions/sr, carrying 69 mJ (0.02% of laser energy) for #16; 3.0 × 10^10^ ions/sr, carrying 17 mJ (0.004% of laser energy) for #29. Higher energies and a larger number of particles are present for #16, corresponding to larger electric-field measurements shown in Fig. 4a. The lower energy-threshold of detection for the spectrometer was ∼1 MeV, and the rapid low-energy decrease of spectra in Fig. 4b is due to this constraint.

### Particle-In-Cell simulations

**Figure 5:**
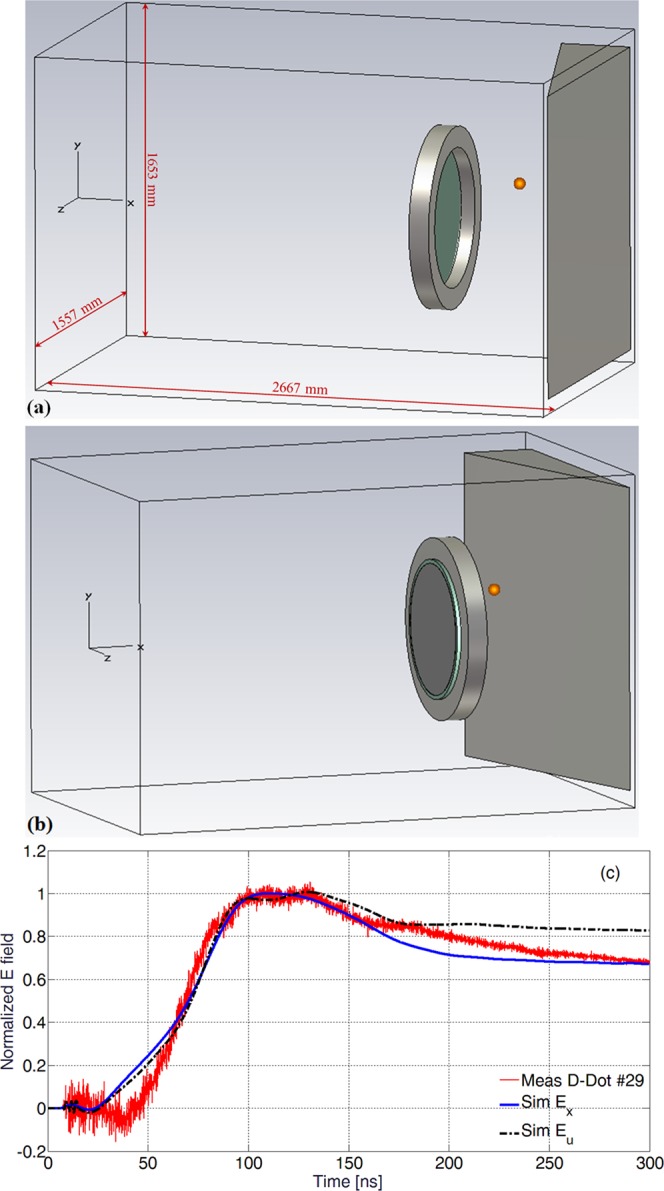
Geometric scheme of the model (**a**,**b**); on both pictures the D-Dot position is indicated by the orange sphere; (**c**) comparison between experimental D-Dot measurements from shot #29 and PIC simulations of E fields at the D-Dot position, in the $\hat{{\boldsymbol{x}}}$ and $\hat{{\boldsymbol{u}}}$ directions.

To have a better description of the electromagnetic field development due to charged-particle dynamics in the setup used in our measurements, we performed proof-of-principle numerical simulations by means of a 3D Particle-In-Cell (PIC) code coupled with a full-wave electromagnetic solver, with details described in the Methods section. The simplified scheme is shown in Fig. 5a,b, where the emission point is indicated by the origin of the *xyz* reference system, the same coordinate as used in Fig. 1. The parabola is modelled as a thin silver layer on a thick glass cylinder, mounted on a stainless-steel annular holder. The tilted stainless-steel trapezoidal object on the right represents a section of the vacuum chamber wall close to the parabola, numerically grounded and conductively connected with the emission point on the front surface. It is well known that different ion species and electron contributions are emitted in this regime of laser-target interaction^1^, but for these simple and preliminary calculations we considered a very basic model, consisting of only one electron and one proton component. In particular, the reasons for using only one keV-range electron component will be discussed in the following part of the manuscript. Emission for both particle species was uniformly distributed within a *θ* = 20° angle to target normal, and also uniform in *β* within the particle kinetic range $[(1-\frac{s}{2}){\beta }_{0};(1+\frac{s}{2}){\beta }_{0}],$ where *β*_0_ = *v*_0_/*c* is normalized particle velocity, with *c* the speed of light in vacuum and *S* = Δ*β*_0_/*β*_0_ the associated velocity spread. According to this model, an optimization process was performed to get a suitable qualitative fit to the experimental data of D-Dot probe in Fig. 3c, together with a reasonable agreement with the particle spectra shown from Thomson Spectrometer results of Fig. 4b. The process involved the velocity ranges of electrons and protons and the relative charges. It led to the determination of the following parameters for the two species:

- protons: *β*_0_ = 0.058, *S* = 60% → (0.774, 2.68) MeV energy; total pulse charge: 35 nC;
- electrons: *β*_0_ = 0.27, *S* = 60% → (9.40, 34.7) keV; total pulse charge: 7.5 nC.

**Figure 6:**
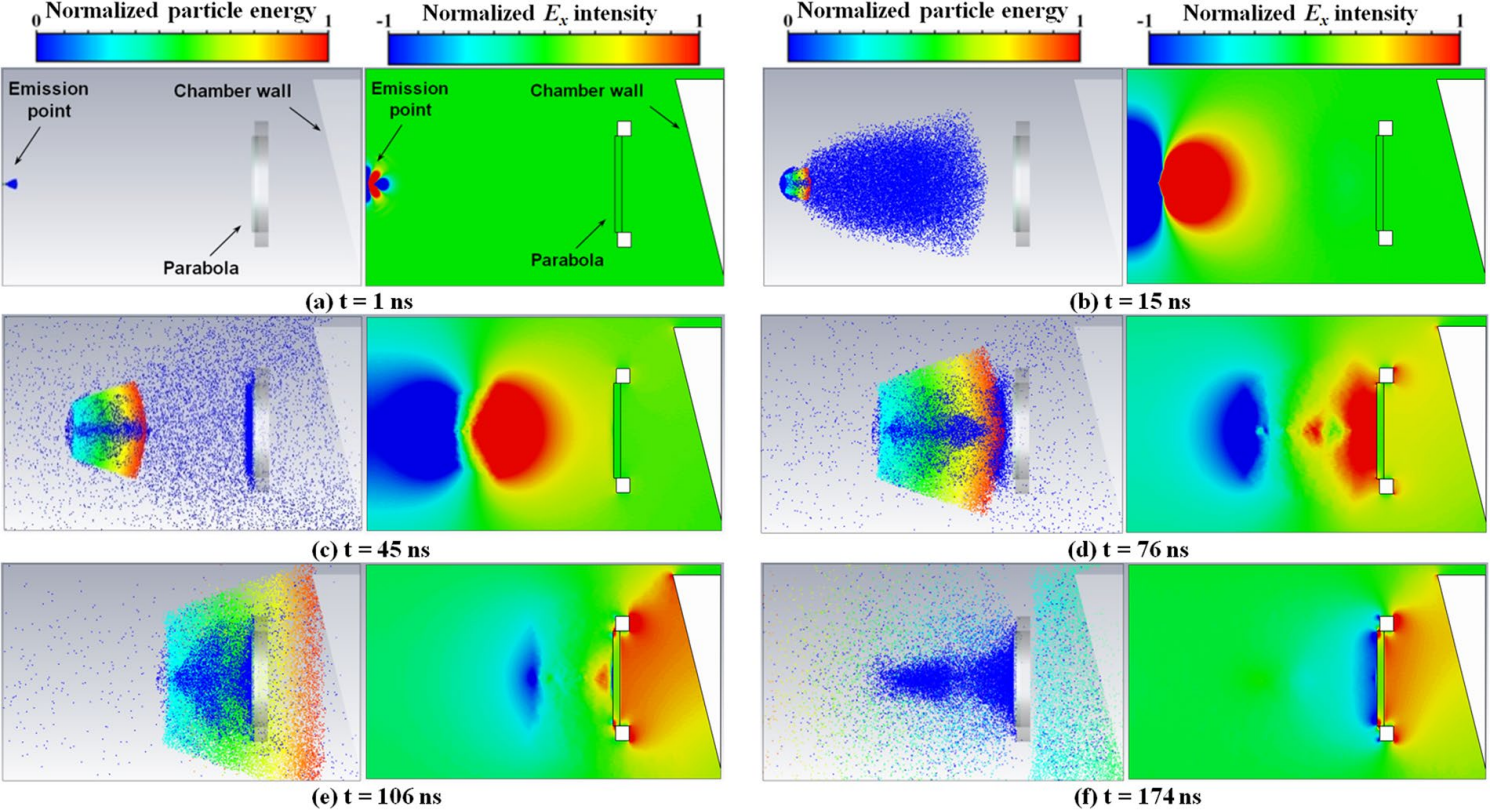
For each time instant, the picture on the left is the top view of particle distribution, and the adjacent one is the *E*_*x*_ field component on the *xy* plane. Particle energy is indicated by the normalized colour scale on the top of the left columns. Electrons are shown with deep blue colour. A normalized scale is used for the *E*_*x*_ intensity and indicated on the top of the right columns. The set of parabolic mirror and annular holder has a maximum transversal extension of 900 mm and is 1800 mm far from the emission point.

We show in Fig. 5c the comparison of the normalized electric field measured for shot #29 by the D-Dot with those obtained via numerical simulations at the same position. In particular, we show both the *x* and *u* (the sensitive D-Dot axis normal to its ground plane, as explained in the Methods section) components of the calculated electric field. Even with this rather simple modeling, a close agreement was reached. Note that in this picture and also in the next Fig. 6, we set the origin of the time scale at the moment of laser-target interaction, and the #29 measurement was thus time-shifted, with respect to Figs 3b and 4a, by the *t*_*s*_ EMP propagation-time from target to D-Dot probe. We found that the optimized proton kinetic energy-range is in rather good correspondence with the most intense part of spectrum measured experimentally by the Thomson spectrometer for #29 and shown in Fig. 4b.

Calculated particle and electric-field dynamics in time are shown in Fig. 6; in particular, the *E*_*x*_ component is displayed on the *xy* plane. After its emission, the ion bunch is accompanied and surrounded by a cloud of electrons having similar velocity (Fig. 6a). Only those electrons with higher energy are capable of escaping the proton attraction and arrive at the parabola before the main charge bunch (see Fig. 6b). In particular, at 16 ns the electron charge propagating with the ions is a small fraction (∼9%) of the proton charge and it is ∼76% of the escaped electron charge. The escaped electrons are responsible for the later rise of the field behind the mirror, which otherwise would be gradually increasing and observable from the moment of ion emission from the target. The primary increase of the electric field at the D-Dot position is due to the proton bunch surrounded by the electron cloud which approaches the parabola, whose effect is observed on simulations to be important for *t* > 30–40 ns (Fig. 6c,d).

When energetic particles reach the parabola surface, secondary electrons are emitted^55^. According to the Furman model for silver^56^, on a neutral surface the *δ* yield of secondary-emitted electrons is higher than one for incoming electrons with energy in the (20,5000) eV range, reaching the mirror for *t* approximately in the (45,680) ns interval. At earlier time, electrons with higher energy, and thus with *δ* < 1, are deposited on the mirror surface. A negatively-charged layer is then generated from the secondary-emitted electrons, typically of low energy (eV level), in close proximity to the associated emitting surface. The negative net charge effectively deposited on the mirror surface is thus lower than the incoming one. The actual number of secondary electrons emitted in a given time instant is also dependent on this effective net charge. For time exceeding 45 ns, *δ* mainly becomes larger than one and there are thus more emitted secondary electrons than incident ones (Fig. 6d–f). The accumulated negative charge on the parabola therefore starts to decrease, eventually becoming inverted in sign. At *t* ∼76 ns the proton beam, accompanied by the low-energy electrons, begins to interact with the mirror and gives an overall positive charge to it (Fig. 6d). Secondary electron-emission due to incoming protons is much lower than that for electrons, because of their different mass and energy^55^. Particles deposited on the parabolic mirror induce a charge also on the trapezoidal section of the chamber shown in Fig. 5, and the parabola and adjacent chamber section then act as a classical capacitor.

In our modeling, the electric field in the D-Dot region increases with time, and reaches a maximum at around 100 ns. For longer times, it appears to be quite stable because of the substantial charge deposited on the parabola (Fig. 6e,f). As suggested by the low slope of the measured temporal profile of the E-field for longer times, the slow neutralization of the residual charge on the parabola would finally cancel the field, and this should take place because of other particles coming at later times and charge relaxation-process through the parabola structure and holder. The fine details of the actual field-intensity distribution on the back side of the parabola will be influenced by its real-world structure, which is not modeled here in detail due to the proof-of-principle nature of these simulations, as discussed in the Methods section; this can be performed more accurately in future works. From simulations, the time behaviour of the field in this region turns out to be rather uniform with respect to the position.

## Discussion

The remarkably large electric-field peaks in the MV/m range as measured by the D-Dot probe, are delayed with respect to the time of laser-matter interaction and are clearly associated with charged particle dynamics. Measurements of spectra for the emitted protons in the backward direction by Thomson Spectrometer TS1 give a suitable indication of this point. The charge up dynamics of the parabolic mirror plus chamber-wall capacitor is indeed very well matched by simple PIC modeling with just one proton and one electron component. This approach is capable of giving good agreement with the measured evolution of electric fields and supplies useful information on their development and distribution in space and time during the parabola-mirror charging process. Of course, more accurate simulations can be performed in future from more detailed modeling of the physical structure, but this lies outside the scope of the work presented here. For this purpose, the use of more particle spectrometers, placed along several directions to target normal, would help to fully characterize particle spectra versus angle, and we plan to implement this in future experimental campaigns.

In experiments of this type, an intense burst of UV, X and γ emission is produced at the moment of laser-target interaction, together with the creation of beams of relativistic electrons with energy-range up to tens of MeVs^1^. The electromagnetic contribution is capable of generating photoionization on any exposed surface, and thus creates a layer of emitted electrons with ∼eV energy surrounding it, leaving a transient superficial *positive* charge. On a slightly longer timescale, MeV-range relativistic electrons are expected to deposit a *negative* charge on the same surfaces since, as observed, secondary-electron emission at those energies is smaller than unity^55,56^. It is currently under investigation, and definitely not trivial, to estimate what the net effect due to the superimposition of these two contrary processes is, in terms of possible associated electric fields. As a matter of fact, these should be detectable during the early moments of the measurements performed by our D-Dot probe (Figs 3c, 4a, 5c), but the contemporary presence on the same detector of the 172 kV/m peak-to-peak field value of the *high-frequency field component* due to classical EMP, might have hidden them in our measurements. This was the reason we considered in our simulations only one low-energy component for the electrons, which was indeed sufficient to give a good phenomenological description of the process observed experimentally. More experimental observations would be needed to fully understand and describe the effects due to the superimposition of these two processes in terms of actual net charge-deposition on a remote surface and related overall field generation along time.

According to previously documented experimental campaigns^32^, the type of experiments we have reported here – several hundred joules, ps laser pulse, intensity higher than 10^20^ W/cm^2^ - is expected to generate the highest values of EMP fields, here identified with the HFC of measured signals, which have one well-recognized generation mechanism due to target charging because of electron emission and the subsequent neutralization current flowing through the target holder^28,29,32^. Indeed, in this work we have shown that, depending on the position within the experimental chamber, and in particular at locations far away from the target, remote LFC fields due to deposited charges can have amplitude approximately seven times higher than EMP fields created by the primary target. In laser-matter interaction experiments, it is very common to use foils or slabs for filtering or completely shielding objects from radiation coming from plasma. As demonstrated here, the deposition of emitted charge on the same filters and shields can induce a very high quasi-electrostatic field, which will be applied to those same objects thought to be protected, and thus might cause severe damaging to their electronics. This is a subtle issue, which should be carefully taken into account when the use of filters and shields is necessary in these contexts. However, these considerations are generally applicable to any region and structure within the chamber, since charge can be deposited on each surface directly exposed to plasma. This is an important result, because such huge fields can be very detrimental for the operation of all the electronic equipment in laser-matter experiments. For this reason the investigation of intense electromagnetic fields is now of great importance and currently a hot topic for present and future plants for laser-plasma acceleration (*PETAL*^57^, *ELI*^58,59^, *Apollon*^60^) and for inertial-confinement-fusion (*NIF*^43,44,61^, *LMJ*^57^).

**Figure 7:**
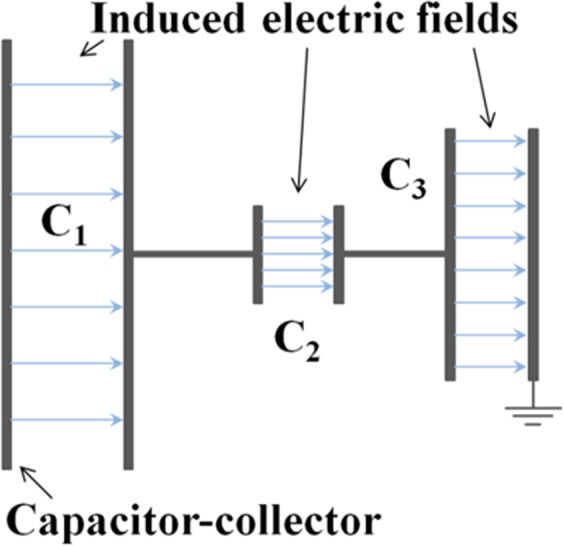
Series connection, to the capacitor-collector, of further capacitors with plates of different surface *S*_*i*_, and associated different *E*_*i*_ fields.

We have described here, for the first time, the sources and evolution in time and space of the large electric fields associated with the charging process of a capacitor-collector structure, due to the large flux of particles emitted and accelerated by powerful laser interaction with matter. This opens up the opportunity to study more complex structures, where different tailored electric-field distributions can be achieved remote from the initial laser-plasma interaction. The electrical field in a classical parallel-plate capacitor scales with the *q*/*S* ratio (being *q* the total deposited charge and *S* the plate surface). If, for instance, a set of open capacitors is connected in series (see Fig. 7), it is possible to deposit the laser-driven charge only on one surface of the first capacitor - acting as an effective charge collector - and then easily create several consecutive regions where the field *E*_*i*_ is differently scaled according to the local plate-surface *S*_*i*_, with *i* capacitor index. In the case of a very small associated surface, extremely-high electric fields might thus be obtained. To make a simple estimation, we can consider to use the 620 mm mirror plate as charge collector, in case of the associated field profiles of several hundreds of kV/m described in Fig. 4a. Thus, in a hypothetical following series capacitor with an electrode having just 20 mm diameter, we might easily reach the order of several hundreds of MV/m electric fields. Several tens of GV/m fields might be also obtained for an electrode of 1 mm diameter. Obviously, the actual limit to the field intensity is given by the breakdown induced in vacuum, which is strictly related to the electrode material, geometry, roughness, conditioning and also to the type and the pressure of the residual gas. However, this breakdown also takes a finite time to develop and begin to neutralize the electric field, holding out the potential for the practical use of these extreme MV/m to GV/m fields over short timescales. Of course, these structures can also be readily designed to obtain non-spatially-uniform field distributions tailored for specific applications, e.g. for control of a laser-accelerated ion bunch.

In summary, the conclusion of our work is that these high fields can be generated in regions suitably-shielded, by means of thick collector plates of high-atomic-number materials, against direct ionizing radiation coming from a laser-matter interaction, that their measurement can be easily and accurately performed by classical conductive field-probes, that the scheme of charge deposition and field generation is versatile, allows for the definition of complex field distributions, and can be efficiently employed with repetitive powerful lasers, for example running at 10 Hz. These electric fields can potentially be used for deflection and energy-selection, in a charged-particle spectrometer-like configuration^34^, of the particle beams emitted by a separate conventional accelerator or one based on laser-plasma interactions. Focusing may also be achieved if charge is conveyed to a classical electrostatic lens^34^. The separation of the two stages - acceleration and conditioning of the beam - is potentially very helpful for the independent optimization of each process, with respect to examples such as those proposed by references^3,7,9^: a large charge might be deposited on the capacitor for effective focusing, regardless of the details of the accelerated bunch. This permits high electric fields and related voltages to be achieved, with short rise-times and at desired time instants absolutely synchronized with the main laser beam, a goal very difficult to obtain with conventional power supplies. This configuration is much more compact and effective for electric beam-conditioning purposes. Once charge is accumulated on the collector, a delayed second laser can be also used to trigger a fast photo-conductively activated *spark-gap switch*^62,63^, short-circuiting the accumulated charge to ground and thus generating very high currents. The technology of these powerful devices allows them to support several tens of kA currents and up to MV voltages with rise times and jitters lower than 100 ps^62–64^. For a reasonable stored charge in the µC range^1,29,32^ peak currents of the order of tens of kA, absolutely synchronized with the second laser, may be achieved. This is the range of currents required, for instance, in a helical-coil structure^9^ to drive an ultrashort travelling electromagnetic pulse, demonstrated to be very effective for controlling and optimizing up to MeV energy laser-accelerated ions. Moreover, very high magnetic fields can be generated with these pulsed currents^20–23,27,33^. These can be effectively used again for particle beam conditioning^4,5,8^ and can be applied also for studies of plasma physics, material science, atomic and molecular physics^20,21^ and laboratory experiments of astrophysical interest^16,33^.

The demonstrated generation of such temporally and spatially controlled fields of high intensity and wide distribution opens up possibilities for the new and significant employment of laser-plasma interaction for *powerful and versatile field sources*, of direct interest not only to particle-acceleration schemes, for which is indeed of primary importance, but also to a multidisciplinary range of applications in different scenarios. To name a few examples, they can be tailored to and very advantageous for biological and medical studies of interactions with cells^35^, material and device characterization^36–38^, EMP-radiation-hardening of components^38^, electromagnetic compatibility studies^38^ and terahertz radiation coming from laser-created plasmas^39,40^.

## Methods

### Experiment description

Experiments were performed using the Vulcan Petawatt laser at the Rutherforth Appleton Laboratory operating at its fundamental wavelength of 1054 nm, for pulses of ∼1 ps duration and focused intensity on target beyond 10^20^ W/cm^2^, as summarized in Table 1. The native contrast was >10^10^ on a nanosecond timescale and >10^6^ at 200 ps before of main pulse, and no plasma mirror system was used here to further enhance the contrast. The thickness of the Parylene-N foil targets used (1.1 g/cm^3^ mass density) is also given in Table 1.

### Electric field probe

A customized version of the AD-80D(R) D-Dot differential electric field sensor^41^ (3-dB-bandwidth up to 5.5 GHz) was placed behind the parabola mirror (see Fig. 2). It was positioned at the (2193 mm, 150 mm, −196 mm) coordinates, for *d*_*DDOT*_ = 2207 mm overall distance from origin, with its sensitive direction (normal to its ground plane): $\hat{u}=0.12\hat{x}+0.87\hat{y}+0.49\hat{z}$. The position and orientation were set for efficient protection against initial direct ionizing radiation due to the laser-matter interaction. The BIB-100G balun (250 KHz–10 GHz bandwidth) was connected to its terminals. The dual structure of the sensor, associated with the balun, allowed for efficient rejection of common-mode disturbance-effects up to more than 28 dB for frequencies up to 6 GHz and even more than 40 dB up to 200 MHz (the frequency range of the Low Frequency Component, as shown in Fig. 3b). A double-shielded RG402 cable ∼25 m long connected the probe to a 12.5 GHz Tektronix DPO71254C oscilloscope, which was used at 50 GS/s acquisition rate per channel, leading to an effective 3 dB bandwidth of ∼6 GHz. The cable-connection was carefully characterized off-line by means of a Vector Network Analyzer Agilent N5230A (10 MHz–20 GHz), and its complex transfer function *A*_*C*_(*f*) was determined in detail. Therefore, from the *V*_*DDOT*_ signal measured on the scope, it was possible to accurately deconvolve that at the balun output:

1 $${V}_{DDOT-B}(t)={{\mathscr{F}}}^{-1}\{{A}_{C}^{-1}(f)\cdot {\mathscr{F}}\{{V}_{DDOT}(t)\}(f)\}(t),$$

where ${\mathscr{F}}$ and ${{\mathscr{F}}}^{-1}$ are the Fourier Transform and Inverse Fourier Transform operators, respectively. The component of the applied electric-field normal to the D-Dot ground plane was recovered by time integration^42^:

2 $${E}_{n}(t)={K}_{DDOT-B}{\int }_{0}^{t}{V}_{DDOT-B}(\tau )d\tau ,$$

being *K*_*DDOT-B*_ = 9.5 × 10^12^ m^−1^s^−1^ a characteristic constant which comprises also the balun attenuation.

A photodiode was used to monitor the time-instant when the laser-pulse hit the target, and was connected to a channel of the same oscilloscope on which the D-Dot probe signal was recorded. The electrical-lengths of both D-Dot and photodiode cable-connections were accurately characterized. In this way we were able to verify, with nanosecond accuracy, that a strong EMP signal was generated at the moment of laser-target interaction, and then reached the D-Dot probe at light velocity after the propagation time *t*_*s*_.

### Measurement accuracy

Oscilloscope resolution and sensitivity limit the minimum value of measured *V*_*DDOT*_ and, as a direct consequence, the accuracy of *E*_*n*_(*t*) that we can obtain from equation (2). In Fig. 3a the acquisition has a time duration much larger than the actual measured signal, and as a matter of fact for *t* > 600 ns we obtained a useful measurement of the scope *background noise*. From the accurate analysis of this time interval, and comparison with acquisitions on null shots where the laser was fired at full energy, but did not actually hit the target, it was possible to estimate the uncertainty on the two components (LFC and HFC) of the reconstructed electric field. In particular, for the LFC field intensity it is *N*_*LFC-FIELD*_ ∼ 20 kV/m, and for the associated field slope *N*_*LFC-SLOPE*_ ∼ 370 V m^−1^ns^−1^. This slope uncertainty is equivalent to a lower observable frequency ∼ 1 MHz. This is clearly not an issue for the HFC, and its intensity uncertainty was estimated: *N*_*HFC-FIELD*_ ∼ 10 kV/m peak-to-peak. In Table 2, the values of the average slopes on the different time-intervals of signal in Fig. 3c are supplied and compared with the related *N*_*LFC-SLOPE*_.

**Table 2:** Average slope for all the intervals for the reconstructed electric field of Fig. 3c and comparison with *N*_*LFC-SLOPE*_.

Interval	Time interval [ns]	|Average slope| [V m^−1^ns^−1^]	|Average slope|/*N*_*LFC-SLOPE*_
a	0–30	992	∼2.7
b	30–93	10011	∼27
c	93–125	74	∼0.2
d	125–167	1987	∼5.3
e	167–266	895	∼2.4
f	266–600	66	∼0.2

We find a very good reconstruction of the original signal slope, with respect to ‘noise’, in the *b* and *d* time intervals show in Fig. 3c; there is still some reasonable signal-to-noise ratio in the *e* interval. Relatively poor accuracy was obtained in intervals *c* and *f* because of the small slope, and also in the *a* interval, but in this case because the average amplitude was only 1.5 times higher than *N*_*LFC-FIELD*_ (see Fig. 3c).

It is well known that the accurate detection of electromagnetic fields by conductive probes is a very delicate and challenging task in experiments with energetic picosecond lasers^33,44,65,66^, especially when the probe is placed inside the vacuum chamber. For this reason, considerable care was taken with cable shielding, and the scope was placed in a separate room, 15 m distant from the vacuum chamber, to suppress direct EMP coupling to it. Possible currents induced on the external conductor of the double-shielded coaxial cables were effectively suppressed by the application, around the cables, of a tailored series of toroids of different materials. We also used a second identical cable for background estimation. This followed the same path to that connected to the balun, was terminated with a 50 Ω load on the vacuum side and connected to another channel of the same scope; thus we verified that any possible EMP coupling to the measurement system, if present, was much lower than the noise level on that channel for those acquisitions.

The D-Dot electric field sensor was placed behind the 110 mm thick, 650 mm diameter parabolic glass mirror, which completely covered it with respect to any direct line-of-sight to the plasma. This ensured good protection from direct particle and ionizing electromagnetic radiation fluxes from plasma. The concern and care about possible measurement artefacts due to these radiations is one of the key-points when performing electromagnetic-field measurements by conductive probes inside the experimental vacuum chamber, because they may otherwise induce spurious current signals on the probes^65,66^. For this reason a dual-structure differential D-Dot probe was here used and coupled with an effective balun. As discussed in the previous paragraph, from specifications^41^ this setup guarantees high rejection of common-mode disturbance effects up to 6 GHz. Detailed discussion on several possible noise contributions is however given in the following section.

We performed Montecarlo simulations, using the SRIM code, of proton ranges within the thick parabolic glass-mirror. These demonstrated that it was capable of stopping up to ∼180 MeV of protons. These energies are much higher than the value of 94 MeV, which is at the moment the maximum documented proton-energy for laser-matter acceleration^67^. Of course, heavier ions have much lower range in the same material. The parabola was thus a very good shield from these possible target-emitted particles.

We also performed further Montecarlo simulations for electron ranges using the CASINO code. In this case the parabola was capable of stopping up to ∼60 MeV energy electrons. In previous TNSA experiments performed with Vulcan laser, electron populations with energies higher than this threshold were reported. As a result we cannot completely exclude the possibility that some relativistic electrons reached the D-Dot probe at the early moments after the laser pulse. However, those electrons would cover the target-D-Dot distance in around the *t*_*s*_ time-interval from the laser shot, and so would arrive very much earlier than the increase of the field measured by the fast D-Dot detector in the interval *b* of Fig. 3c. Moreover, a classical TNSA electron spectrum decreases rapidly with energy, and thus only a very limited amount of electrons with that very high energy should have reached the probe. In Figs 3c and 4a no relevant associated signal was actually observed in the first ten nanoseconds. This demonstrates that if any common-mode contribution occurred, it was effectively rejected by the dual D-Dot-balun configuration, generating a negligible contribution to the measurement, which might be also hidden by the simultaneous presence on the same detector in that interval of the 172 kV/m peak-to-peak field value of the high-frequency field component (HFC) due to classical EMP.

Similar considerations apply for the UV-X-γ radiation emitted from the target. It is well known that in this type of laser-matter experiments the typical emitted spectral density for this electromagnetic contribution decreases with the associated photon energy^1,19,25,26^. For this reason the largest part of this flux would be absorbed by the thick glass-parabola, and those γ-rays possibly energetic-enough to pass-through it, would of necessity then have a low cross-section of interaction with the D-Dot probe. These should have arrived at the D-Dot within the first few nanoseconds after the laser pulse, and thus well within the *a* interval of Fig. 3c and Table 1: very much earlier than the field increase in the interval *b* of Fig. 3c. But, again, no signature for a possible effect of this type was clearly visible on the measurements of Figs 3c and 4a, possibly due to the high rejection of the probe to common-mode effects and to the superimposition on the same probe of the high-frequency field component due to classical EMP.

A background radiation contribution may also come from bremsstrahlung of accelerated particles hitting the surfaces of the chamber and/or those of objects present within it. It is well known that the most intense contribution should be due to electrons, and increase with their energy^19,25,26^. This should have produced a background common-mode contribution in the chamber mostly due to fast electrons, and thus be present during the first tens of nanoseconds from the laser pulse, so again amply within the *a* interval of Fig. 3c and Table 1. But, as in the previous cases, this was not observed for Figs 3c and 4a, possibly due to the contemporary presence of the EMP on the same detector, and to its high rejection to common-mode disturbances.

Accelerated particles can induce secondary electron emission when interacting with any surface in the chamber^55^. This is mostly due to incoming electrons. Secondary-emitted electrons have energies of the order of a few eV and a related maximum production for incoming electrons up to a few keV energies, being the secondary-electron-yield decreasing for higher energies. The D-Dot probe was far (a minimum of 30 cm) from any surface directly intersected by particles coming from the laser-matter interaction. As a rough estimation, an electron with 1 eV energy would cover the 30 cm distance in more than 500 ns. This is much later than the *a, b, c, d, e* time intervals described in Table 2 and shown in Fig. 3c, and might effect, in such a case, only the *f* interval, for which we have anyway pour measurement accuracy (see Table 2).

We can conclude that the present campaign provided a very good measurement robustness against possible spurious signals from ionizing radiation and related secondary effects present within the experimental chamber for the D-Dot probe, at least in the *b*, *c*, *d*, *e* time-intervals.

### Thomson spectrometer

The Thomson Spectrometer TS1 was placed 39 cm from the foil target, at an angle of 69° with respect to the $\hat{x}$ direction. Magnetic deflection was performed over a 50 mm length, with an applied maximum static magnetic field of 0.54 T. Electrostatic deflection was achieved in a tapered-electrode structure of similar height with a plate length of 200 mm, minimum and maximum plate separation of 2.5 mm and 20 mm, respectively, for an applied potential difference of 6250 V. Imaging Plate BAS-TR was employed as an ion detector, and associated calibration data for protons^68,69^ were used to obtain the spectra shown in Fig. 4. For the estimation of the detection noise-level, a section of the imaging plate was taken as close as possible to the proton parabola line, and from it the associated noise level σ was obtained. An arbitrary non-zero detection level of 2*σ* is here considered for suitable data accuracy, and shown for shots #16 and #29 in Fig. 4b. The lower energy-threshold of ∼ 1 MeV observed in Fig. 4b is due to the geometrical constraints of the spectrometer. The Spectrometers TS2 and TS3 in Fig. 2a were at ∼1.5 m distance from target. TS2 was normal to the target and TS3 at an angle of ∼10° with respect to the normal.

### PIC simulations

Simulations were performed using the CST Particle Studio^®^ code. This particle-in-cell solver can perform fully-self-consistent simulations of particles and electromagnetic fields in the time domain. Fields are calculated by the Finite Integration Technique^70^ which is applied to the integral form of Maxwell’s equations in time domain. In these simulations the focusing parabola is placed 1800 mm from the emission point, and modelled as a thin silver layer of 620 mm diameter on a 110 mm thick glass cylinder of 650 mm diameter, mounted on a 100 mm-thick stainless-steel annular holder with a 900 mm external diameter.

Space-charge effects were also computed in the simulation, together with secondary electron emission^57^ and superficial charge deposition on all surfaces. Here we are not interested in the acceleration process during the laser-matter interaction, but rather in the fields associated with and produced on surfaces by particles when they are rather far from the emission zone. This allows for the use of more relaxed conditions in simulations. Each particle specie was modeled with the same Gaussian time-profile $A{e}^{-\frac{{t}^{2}}{2{\tau }^{2}}}$, having maximum emission at the initial time of the simulation *t* = 0, with a *τ* = 0.5 ns inflection point with respect to the maximum. The bunch time-dependence is slightly modified from the classical Gaussian as to be zero at *t* = 1 ns. We kept the overall bunch charge to low values to minimize space-charge effects, causing excessive enlargement of the actual beam divergence with respect to the 20° setting, and thus mainly leading to fewer particles intercepting the far parabolic mirror. The emission point is modelled as a circular surface of 1 mm radius on the *yz* plane, divided into 1521 sub-surfaces of equal emission properties and uniform charge density. In particular, the density was *σ*_*p*_ = 11.14 × 10^−3^ C/m^2^ for protons and *σ*_*e*_ = 2.39 × 10^−3^ C/m^2^ for electrons. The actual time step used for stable and accurate simulation in our case was 4.75 ps. The simulation box dimensions were 2667 mm, 1653 mm and 1557 mm along *x*, *y* and *z* axes, respectively. A maximum frequency of 1 GHz was set for the algorithm of λ-based adaptive mesh refinement. Twenty lines per wavelength were used as a general indication, and the local mesh distance was changed according to the shape and local dimension of modelled objects, for a total number of about 7.5 × 10^6^ mesh cells. Minimum and maximum mesh step sizes were 1 mm and 17.05 mm, respectively.

In these proof-of-principle simulations we dealt with the phenomenological modeling of the parabola charging, and so a simple geometric scheme was considered; indeed, as shown in Fig. 5c, this was able to give an effective representation of the field evolution observed experimentally, with results in rather fine agreement with normalized laboratory measurements. This simple model is less appropriate if we seek to reproduce the actual maximum field of the measurements. In this case it is necessary to describe also the acceleration process and to find the correctly tailored proportion of multiple electron and ion populations from the moment of emission^1^. Moreover, the field is not only due to deposited charge but also influenced by the precise configuration of the setup we used in the vacuum chamber. More accurate field descriptions thus require access to detailed data on the exact configuration, precise mechanical details and materials of the mirror support structure and also of each object present within the experimental chamber, especially those next to the parabola. In a typical experiment, objects are moved on a shot-to-shot basis, and thus the model should be updated accordingly. This is a very demanding task, not only for the remarkable work of modeling needed for these multi-scale extensive simulations, especially for the large experimental chamber used on Vulcan facility, but also for the computational-time and memory-resources required, and we think it is out of the scope of the present paper. Nevertheless, to estimate what might be expected from more realistic representations, we performed further simulations with more-detailed models of the parabola support and of its connection with the floor of the experimental chamber.

These simulations showed that the presence of sharp corners or surfaces on the back-side of the parabola holder, close to the D-Dot position, can locally increase the field at that point for a given deposited charge, as expected. A few orders-of-magnitudes of field-increase might be possible. It is well-known that the field increment due to an edge depends on its geometry and materials used, and also on the very precise modeling of the surrounding objects. Indeed, these simulations showed that the time-evolution of fields was in general not affected significantly, because the process is mainly driven by particle deposition and secondary emission on the parabola-mirror surface rather than edge effects.

The results obtained for these more-accurate models showed that if the parabola supporting structure is short-circuited by a good conductor to the metallic floor of the chamber, the field time-profile and rise observed in Fig. 5c appear amplitude-modulated by oscillations of the megahertz order, due to the creation in the chamber of resonating modal-fields. These are triggered by the current flowing through the parabola support because of charge accumulated on the silver-plated glass, similarly to what happens for the classical model of EMP generation due to neutralization current through the target holder^29,32^. These oscillations have an amplitude dependent on the mechanical details and on the materials used for the equivalent short-circuit, and on the overall geometrical configuration of the chamber and of each object placed within it. In other words, on the equivalent complex-impedance connected with the parabola holder. The resulting field profile is somehow different to that observed in Figs 3c, 4a, 5c, even if the envelope of the overall signal remains in line with the simulation and measurement results observed in those Figures. In conclusion, this configuration is not capable of providing a suitable phenomenological reproduction of the process observed experimentally. If, instead, a perfect-dielectric or, similarly, a highly-dissipative connection is used in the model to link the parabola support to the floor, we observe that those oscillations disappear, and the time-profile results are generally very similar to those seen in Fig. 5c. This configuration is thus found to be more appropriate to represent the observed measurements phenomenologically, and when the perfect-dielectric considered is vacuum, indeed we come to the simple model shown in Fig. 5.

In conclusion, the simple model described in this paper was found to be suitable to give a proper phenomenological representation of the process observed experimentally. Improved designs can be implemented to get more accurate results, but the complexity of these multi-scale extensive numerical studies makes their description, in our opinion, beyond the scope of the present paper and more appropriate for later evolutions of this work.
